# Knowledge and perceptions of obesity care among general practice nurses in the Republic of Ireland

**DOI:** 10.1017/S1463423626101285

**Published:** 2026-07-28

**Authors:** Ciara Donaghue, Roisin Doogue, Suzanne Seery, Joanne Cecil, Francis M. Finucane

**Affiliations:** 1 Centre for Obesity and Metabolic Health, Connolly Hospital, Dublin, Ireland; 2 Department of Medicine, College of Medicine, Nursing and Health Sciences, University of Galway, Galway, Ireland; 3 School of Medicine, University of Limerick, Limerick, Ireland; 4 HSE, National Clinical Programme for Obesity, Dublin, Ireland; 5 School of Medicine, University of St Andrews, Scotland, UK; 6 Cúram, University of Galway, Galway, Ireland; 7 Galway University Hospitals, Galway, Ireland

**Keywords:** barriers, general practice nurse, knowledge, obesity, perception, primary care, training

## Abstract

**Aims:**

We sought to explore the knowledge and perceptions of obesity care among general practice nurses in the Republic of Ireland.

**Background:**

Obesity is a complex condition driven by the interplay of social, cultural, environmental, genetic, biological and psychological factors. Despite its recognition as a chronic disease, it is underdiagnosed and undertreated in primary care. General practice nurses (GPNs) play a critical role in the monitoring of risk factors and treatment of chronic diseases, but their role in the management of obesity in primary care is less well described.

**Method:**

A cross-sectional cohort study was conducted. An anonymous online survey was disseminated asking a broad range of questions on levels of training, knowledge, perceptions and barriers to obesity care.

**Findings:**

Of 240 GPNs who completed the survey, 93% were aware that obesity aetiology is multifactorial. Identified causes which imply “personal responsibility” rated highly (73%). Most GPNs are comfortable having discussions about weight (74%). They reported discussing behavioural interventions more frequently than pharmacological treatments or bariatric surgery. Perceived lack of time in appointments (83%), lack of training (71%), shortage of specialist care (72%) and cost of treatments (67%) were the highest rated barriers to providing care. Lack of time in appointments (67%) and fear of causing offence (57%) were the highest rated barriers to initiating a discussion with patients about weight. GPNs felt their own BMI influenced how patients with obesity view their advice. Many GPNs (40%) reported they had no training in obesity care, while 54% had attended at least one obesity-themed webinar or conference.

**Conclusion:**

GPNs recognise their essential role in obesity care. We have identified the need for dedicated consultation time and further training for GPNs to enable discussion of treatment options and support appropriate care pathways, including access to specialist care.

## Introduction

As in other countries, obesity in Ireland poses a major burden on affected individuals and on healthcare systems, with direct healthcare costs of obesity-related conditions predicted to reach more than €5 billion by 2030 (Keaver *et al*., 2013; Donovan and McNulty, 2024). Primary care is usually the entry point for referral to specialist services (Busetto, 2024; O’Connor *et al*., 2025). For patients seeking to discuss weight-related health concerns within general practice, there are significant opportunities to help manage obesity and prevent its complications (O’Donoghue *et al*., 2021; Crotty *et al*., 2022).

General practice nurses (GPNs) are privately employed by general practitioners (GPs) in Ireland. They contribute to the delivery of services aligned with public health policy and to meeting contractual obligations within general practice. They provide holistic healthcare and play a critical role in nurse-led assessments, chronic disease management, and preventive care (Irish General Practice Nurse Educational Association, 2024). They work both independently and in collaboration with GP’s and community teams. GPNs understand the cultural, social and economic environment, family situations, living habits and the local community of patients within their practice (Bornhoeft, 2018; Yu *et al*., 2021). A recent general practice survey in Ireland noted 13,161 nurse consultations nationally in a single day (Collins and Homeniuk, 2021).

Under the Model of Care for the Management of Overweight and Obesity (Model of Care), primary care is tasked with early identification of obesity and related complications, brief advice on the condition, initial management and signposting to specialist services (Health Service Executive, 2021). GPNs are encouraged to complete the ‘Make Every Contact Count’ learning module (MECC) on the topic ‘Talking about Overweight and Obesity’ to help them support patients through providing non-stigmatising care (Gaynor *et al*., 2023). The Association for the Study of Obesity in Ireland (ASOI) Clinical Practice Guidelines on the Management of Obesity in Adults (CPGs) provide recommendations for care underpinned by evidence-based principles of chronic disease management (Breen *et al*., 2022). These resources are accessible to all healthcare professionals (HCPs) including primary care practitioners (GP’s and GPNs) and allied health professionals in Ireland.

Primary care faces many challenges in delivering evidence-based obesity management. Lack of training, lack of consultation time, competing clinical demands, deficiency of referral pathways and costs of treatments are previously described barriers to care (McHale *et al*., 2020; Oshman *et al*., 2023). In the UK, GPNs supported more people and spent more contracted hours on obesity management than other primary care nurses, despite a lack of specific training or organisational support (Brown *et al*., 2007). We sought to gain insights from GPNs by determining their perceived current level of education and training, their perceived knowledge of the causes of obesity, perceptions of obesity care and perceived barriers to providing care. These findings could support the planning of education and training resources where gaps are identified.

## Methods

### Participants

GPNs practising in the Republic of Ireland were invited to participate in the study. The Nursing and Midwifery Board of Ireland, State of the Register Report, identified 2318 GPNs working in Irish general practice (Nursing and Midwifery Board of Ireland, 2022). Multiple methods of questionnaire distribution were used to invite these GPNs to complete our survey. GPNs were contacted through the Irish General Practice Nurse Educational Association and then also by the Professional Development Coordinators for General Practice Nurses via email, in addition to regular posts on social media platforms. We did not record the number of GPNs invited through each of these three domains, but invitations were circulated as widely as possible. Survey data were collected anonymously, with no personally identifiable information recorded from any respondent.

### Questionnaire

The survey design enabled a broad spectrum of questions to be asked and was conducted using a Microsoft Forms questionnaire (see supplementary material). The survey was adapted from two other surveys used in similar studies: The Causal Attributions for Obesity Scale and The Communication on Overweight and Obesity Project Questionnaire (Pearl *et al*., 2018; Laidlaw *et al*., 2019). Two questions were added to reflect the ASOI CPGs. The survey was pre-tested by a pilot group of GPNs, and two changes were made. We included an explanation of “eating out of home,” and we amended the term practice nurse to nurse working in general practice, to include advanced nurse practitioners. The final questionnaire comprised of 20 questions and took approximately 14 minutes to complete (see supplementary material). The anonymity of this design allowed respondents to give honest, confidential responses to the questions. The survey measured: (i) demographics; (ii) perceived level of training in obesity management; (iii) perceived knowledge of the causes of obesity; (iv) perceptions of GPNs on raising the topic about weight and providing care; (v) the perceived role of the GPN in assessment and treatment of people with obesity; (vi) barriers to providing obesity care. There was an optional question asking GPNs to provide their own body mass index (BMI) as studies have shown that a nurse’s own weight can influence their provision of obesity care (Jeffers *et al*., 2024).

### Data analysis

Descriptive analyses were performed using IBM Statistical Package for Social Sciences. The chi-square statistical test was used to determine differences in proportions between categorical variables. We merged participants with a BMI ≤ 24.9 kgm^‒2^, participants with a BMI of ≥ 25–29.9 kgm^‒2^, and those with a BMI ≥ 30 kgm^‒2^, into three distinct categories.

## Results

### Sample

Of the approximately 2,138 potential participants contacted via email by the IGPNEA and practice development coordinators, 241 GPNs completed the survey. One was excluded because they did not work in general practice, leaving 240 participants. Self-reported BMI data were available for 234 of these respondents.

### Demographics

The age range for participants was 25–72 years. The level of professional experience varied: 27% had less than 5 years’ experience, 24% had between five and 10-years’ experience, and 49% had more than 10 years’ experience. All, bar one GPN, were registered general nurses and 45% had dual qualifications (such as midwifery) or an expanded role (nurse prescriber or advanced nurse practitioner), or both. Self-reported obesity was present in 24% of participants while an additional 33% were overweight (see supplementary material).

### Education on obesity care

**Figure 1.**
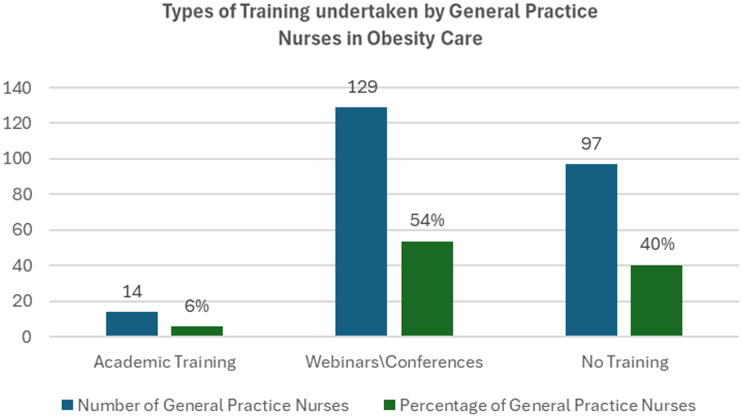
Levels of training.

Six percent of GPNs reported having academic training in obesity care; 54% had attended obesity-themed webinars or conferences, while 40% described having ‘no training’ (Figure 1). GPNs were asked if they had ‘accessed’, ‘not accessed’ or were ‘unaware’ of the Irish Health Service’s ‘Making Every Contact Count‘ module, and 65% of GPNs had not completed or were unaware of it. Only 16% of GPNs were aware of and had accessed the ASOI’s clinical practice guidelines.

### Knowledge of causes of obesity

GPNs rated the importance of a number of causes of the development of obesity (Table 1). Most GPNs rated biological and environmental causes of obesity as either important or very important. Personal responsibility (laziness and lack of willpower) was rated as either ‘important’ or ‘very important’ by 73% of GPNs (Table 1). The perceived importance of laziness was lower in GPNs with training in obesity, but there was no difference in the perceived importance of lack of willpower according to training level, as shown in Table 2.

**Table 1.** 240 general practice nurses, in the Republic of Ireland, rating of the importance of individual causes of the development and/or progression of obesity, adapted from the Causal attributions for obesity scale (2024) Table 1 long description.

	Not important *N* (%)	Important *N* (%)	Very important *N* (%)
**Brain/Biology**			
Genetic factors	12 (6)	139 (58)	89 (37)
Metabolic factors	6 (6)	107 (45)	127 (53)
Endocrine disorder	8 (3)	102 (43)	130 (54)
Addictive properties of food	8 (3)	91 (38)	141 (59
Psychological/emotional problems	2 (1)	91 (38)	147 (61)
Stress	5 (2)	113 (47)	112 (51)
Poor quality or lack of sleep	8 (3)	104 (43)	128 (53)
**Personal Responsibility**			
Lack of willpower	44 (18)	110 (46)	86 (36)
Laziness	86 (36)	105 (44)	49 (20)
**Behaviour**			
Not enough physical activity	10 (4)	118 (49)	112 (47)
Overeating	7 (3)	112 (47)	121 (50
Eating out of the home, eg shops, takeaways, restaurants	17 (7)	112 (47)	111 (46)
**Environment**			
Poor nutritional knowledge	9 (4)	91 (38)	140 (58)
Marketing of unhealthy foods	15 (6)	99 (41)	126 (53)
Low income	25 (10)	110 (46)	105 (44)

**Table 2.** Chi-square testing statistical analysis results of questions asked to general practice nurses on their knowledge and perception of obesity care among general practice nurses in the Republic of Ireland (2024) Table 2 long description.

Question	*P*-value	Result
Does having previous training make it more likely that general practice nurses accessed the MECC module?	0.002	Yes
Does having previous training make it more likely that general practice nurses accessed the ASOI Guidelines?	0.001	Yes
Does training influence general practice nurses rating of the importance of laziness in the development and/or progression of obesity?	0.024	Yes
Does training influence general practice nurses rating of the importance of laziness in the development and/or progression of obesity?	0.080	No
Does the BMI of a general practice nurse influence how they rate laziness?	0.430	No
Does the BMI of a general practice nurse influence how they rate lack of will power?	0.379	No
Do general practice nurses feel their BMI affects how patients with obesity view their advice?	0.001	Yes

Five GPNs with a BMI ≥ 40kgm^‒2^ all rated laziness as ‘not important’, whereas of 103 GPNs with a normal BMI only 34 rated laziness as ‘not important’, suggesting influence of a GPN’s BMI on their rating of the importance of laziness. However, due to small sample sizes, BMI categories were merged in 3 categories, normal, overweight and obesity, and GPN’s rating of laziness was then statistically not depending on BMI (Table 2).

### Perceptions in raising the topic of weight and providing obesity care

When initiating a discussion with patients about their weight, which 64% of GPNs reported doing, the main barriers to doing so were ‘lack of time in appointments’ (67%), ‘fear of causing offence’ (57%) and patients ‘attending for a different reason’ (56%). GPNs agreed that they have ‘an essential role in assessing and treating people for obesity’ (96%), and 90% believed they can ‘have an effect on a patient’s ability to lose weight’. Most GPNs disagreed that ‘treatment for weight loss should only be offered to adults living with obesity (not overweight)’ and 95% agreed that ‘for people living with obesity even small weight loss can produce health benefits’. While 40% of GPNs with a normal BMI felt that their weight influenced how patients with obesity perceived their advice, this proportion rose to 85% among those with a BMI ≥ 30 kgm^‒2^.

### Assessment and treatment interventions

**Figure 2.**
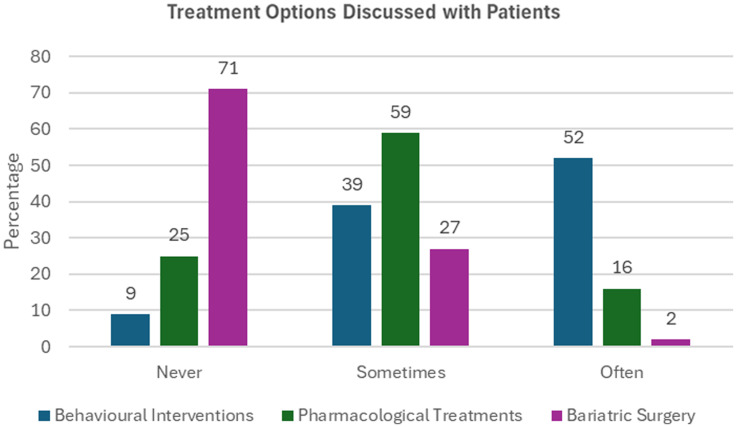
Treatment options discussed.

Body mass index was the most frequently used method of assessment (97%). Waist measurements were taken by 79% of GPNs, and 6% used the Edmonton or Kings Obesity Staging Criteria (Padwal *et al*., 2011; Valderhaug *et al*., 2016). Behavioural lifestyle modification interventions were discussed by 91% of GPNs but only 29% reported discussing bariatric surgical interventions with patients with obesity. Pharmacological treatments were discussed by 75% of GPNs but by 95% of nurse prescribers (Figure 2). Pharmacological treatments were most frequently discussed ‘if a patient requested it’ (83%).

### Barriers to care and conversations

**Figure 3.**
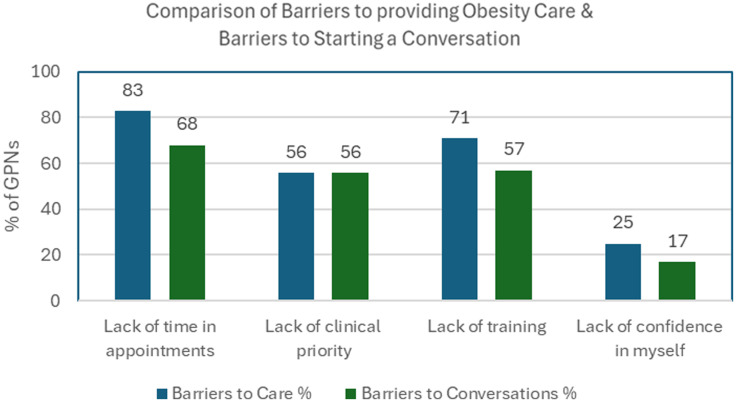
Barriers to care and conversations.

‘Lack of time in appointments’ was the highest rated barrier to care (83%) (Figure 3). ‘Shortage of specialists or specialist programmes to refer people to’ and ‘the cost of treatment’ were barriers selected by 72% and 66% of GPNs respectively. ‘Lack of training to discuss obesity management’ was chosen by 71% of respondents (Figure 3). Resources that would help GPNs care for people living with obesity aligned with barriers experienced and included ‘access to obesity management programmes’ (68%) and ‘increased education related to the management of obesity’ (57%).

## Discussion

This study aimed to identify GPN’s knowledge and perceptions of the causes and management of obesity in general practice in Ireland. We found that GPNs recognise the multifactorial nature of obesity, are confident discussing weight with their patients and perceive their role as important. Treatment focus is on behavioural interventions and pharmacological therapies. However, many GPNs feel underprepared and lack confidence in the care and management of this chronic disease. They recognise the educational, organisational and systemic barriers to providing high-quality, evidence-based care. These findings are consistent with those from studies in other jurisdictions (Caterson *et al*., 2019; McHale *et al*., 2020).

Our study found that most GPNs feel inadequately prepared to manage patients with obesity. Many have no training in obesity care and are unaware of available resources. GPNs who participated in webinars on obesity care were less likely to attribute obesity to personal responsibility and demonstrated increased engagement with educational resources, suggesting that webinars may serve as an effective modality for professional training. In Ireland, access to funded education for GPNs is limited compared to nurses working in other clinical areas (McCarthy *et al*., 2012). Lack of financial support, educational leave, and organisational support can prevent access to training (Bornhoeft, 2018; Oshman *et al*., 2023; O’Shea and Doogue, 2024). Professional bodies have called for continuing medical education for primary care practitioners to address training gaps, but this will require funding (Crotty *et al*., 2022).

This study indicates that GPNs recognise the biological causes of obesity and the environmental influences on its development. However, ‘personal responsibility’ and ‘behaviours’ related to ‘eating and physical activity’ also rated highly as causes of obesity. This aligns with the stigmatising narrative of obesity as an individual’s lifestyle choice (Brown *et al*., 2007; Puhl and Heuer, 2009; O’Donoghue *et al*., 2021). It may also reflect the coexistence of biomedical and behavioural frameworks commonly used in clinical assessment.

We found that that BMI is the primary assessment tool used by GPNs, rather than more sophisticated and potentially informative clinical staging tools, such as the Edmonton Obesity Staging System (Padwal *et al*., 2011). Adopting these could complement nursing frameworks and guide comprehensive assessment by considering psychological, functional, and medical impairments associated with obesity. Assessment results can be used to guide treatment goals in clinical practice (Padwal *et al*., 2011; Busetto, 2024; Sen *et al*., 2025). Our findings highlight the need for further education on the causes obesity and better implementation of evidence-based guidelines for obesity care (O’Donoghue *et al*., 2021; Breen *et al*., 2022; National Institute for Health and Care Excellence, 2025).

The Irish Model of Care identifies primary care as having a critical role in screening, assessment and referral to specialist services (Health Service Executive, 2021). GPNs in this study agree they have an essential role in identifying and treating patients with obesity but lack of clinical priority and lack of time in appointments can result in other work being prioritised. A UK study of primary care clinicians highlighted the importance of treating the patient’s presenting complaint first and expressed concern about deviating from the patient’s agenda (Blackburn *et al*., 2015). Compared with other chronic conditions, care for patients with obesity has been under-resourced (Health Service Executive, 2021; Crotty *et al*., 2022). The Irish Chronic Disease Management Programme, introduced into primary care in 2020, has led to improved health outcomes for patients (O’Connor *et al*., 2025). The addition of obesity to this programme could have positive benefits by providing dedicated time and prioritising obesity care during structured consultations. Organisational focus on education of GPNs supported by structured care pathways and dedicated appointment times may address some of the barriers to care identified (Jeffers *et al*., 2024; Curran *et al*., 2025).

Historically, obesity care focused on behaviours as treatment, rather than addressing the causes of obesity beyond an individual’s control (World Health Organisation, 2023). The landscape of obesity care is changing, and the choice, type and intensity of treatment should be based on individualised therapeutic goals and the clinical severity and stage of obesity, taking into account available therapeutic options, possible side effects and patient preferences (Brown *et al*., 2007; Crotty *et al*., 2022; Busetto, 2024).

While treatment options discussed depend on the severity of obesity, lifestyle modification is the cornerstone of the therapeutic approach to care (Crowe *et al*., 2018). This is reflected in the finding that behavioural structured lifestyle modification interventions were the treatment option most often discussed by GPNs. International guidelines recommend multi-component behavioural interventions, but these are difficult to implement in general practice in Ireland due to time constraints and limited access to multidisciplinary teams (Semlitsch *et al*., 2019). This may explain why GPNs identified improved referral pathways and access to obesity management programmes as resources that would help them support their patients.

Pharmacotherapy is recommended as an adjunct to behavioural interventions in long-term management of obesity (Breen *et al*., 2022). Pharmacological treatments were frequently discussed by GPNs, suggesting growing awareness of their role in obesity management. A European study found that many HCPs remain unfamiliar with obesity medications; some suggest they should be prescribed in primary care (Rubino *et al*., 2021). Cost of medications can be a significant barrier to pharmacotherapy in Ireland, limiting patients’ access and influencing prescribing decisions (Sumithran *et al*., 2023). We found that GPNs are more likely to discuss pharmacotherapy when patients request support, emphasising the importance of a strong therapeutic relationship.

Bariatric surgery is the most effective treatment for severe and complex obesity (Fearon and Heneghan, 2022). Current service provision in Ireland does not meet existing demand, with Ireland performing fewer surgeries than many other European nations (Craig *et al*., 2018). HCP’s in Canada viewed bariatric surgery as a ‘last resort’ due to limited accessibility and strenuous pre-surgical work-up (Aboueid *et al*., 2018). Our findings show that GPNs discuss bariatric surgery least among treatment options. These findings suggest that barriers to accessing obesity care in Ireland may reflect the broader systemic, economic and political challenges faced worldwide (Curran *et al*., 2025).

Most GPNs reported that they are comfortable discussing weight with their patients. However, lack of training and lack of confidence were highlighted as barriers to those discussions. Discordance has been observed between self-reported practice and observed practice (Laidlaw *et al*., 2019). In one Irish study, HCPs said their patients started the discussion of obesity 43% of the time, whereas; 59% of patients with obesity in the same study report starting the discussion (Curran *et al*., 2025). Patients with obesity want their HCP to initiate the discussion (Caterson *et al*., 2019; Curran *et al*., 2025).

GPNs lack of confidence about discussing obesity with patients may be due to uncertainty about the credibility of their message and reciprocal judgements between patients and HCPs based on physical appearance (Blackburn *et al*., 2015; Jeffers *et al*., 2024). Half of all GPNs, in our study; and 85% of those with obesity indicated that their weight affects how patients with obesity view their advice. This complex problem is unique to weight-loss consultations (Phillips *et al*., 2014). Nurses with a low BMI are concerned they appear to lack empathy, while nurses with a high BMI are concerned about being poor role models (Jeffers *et al*., 2024; Laidlaw *et al*., 2019). To address these perceived credibility concerns, we think that GPN training programmes should include stigma awareness and reflective practice. This would provide opportunities for GPNs to explore the extent to which personal experience influences their consultations (Blackburn *et al*., 2015; O’Donoghue *et al*., 2021; National Institute for Health and Care Excellence, 2025). Training in communication skills and structured frameworks such as the 5A’s approach (Ask, Assess, Advise, Agree, Assist) may enhance GPN’s confidence in the delivery of their message and facilitate more effective, patient-centred care (O’Shea *et al*., 2021; Crotty *et al*., 2022; National Institute for Health and Care Excellence, 2025).

## Recommendations

Access for GPNs to funded education that addresses gaps in knowledge of evidence-based practice, the complexity of obesity and the impact of weight stigma.

Improved visibility and availability of obesity treatment related resources.

Dedicated resourcing of obesity management within the structure of the Chronic Disease Management Programme.

## Limitations

A limitation of this study is the absence of precise data on the number of individuals who were invited by email to participate. However, it is likely that the vast majority of the 2,318 GPNs in Ireland received an email invitation, were informed about the study and had the opportunity to participate. The response rate of 240 GPNs, approximately 10%, is relatively low and may have introduced bias and limited the generalisability of our findings to all GPNs in Ireland. The online anonymous survey might only reflect the opinion of those with a greater interest in obesity care. The questions posed did not allow for objective evaluation of academic achievement or formal clinical training undertaken. We did not ask GPNs to consider the severity of obesity when discussing various treatment options. Future research should address these limitations.

## Conclusion

We have found that GPNs understand and appreciate the multifactorial causes of obesity. They recognise their essential role in the identification, treatment and management of obesity, but less than one third feel adequately prepared for this task. They report barriers to providing accessible evidence-based care at general practice level, including limited time in consultations, lack of training on obesity management, shortage of specialist services and the high cost of treatment. Action is required to overcome these barriers to enhance accessibility to care for patients with obesity in general practice.

There is a need for collaborative education and care between GPNs and patients with obesity to promote an understanding of obesity and reduce weight bias and stigma in primary care. Despite the challenges there is much to be optimistic about. Obesity is recognised as a chronic disease; new treatments are becoming increasingly available, and investment is being made to implement the Model of Care (Health Service Executive, 2021). GPNs and the primary care setting are the gateway to obesity care for most patients. Equipping GPNs with the skills and resources to identify and support those who require this care is critical to obesity management in the future.

## Supporting information

10.1017/S1463423626101285.sm001 Donaghue et al. supplementary material Donaghue et al. supplementary material

## Data Availability

Data are available upon reasonable request.

## Supplementary material

The supplementary material for this article can be found at https://doi.org/10.1017/S1463423626101285.

## Table 1. Long description

A table showing the importance ratings of various causes of obesity by general practice nurses. The table has 4 columns: Not important (N %), Important (N %), and Very important (N %). The rows are grouped into four categories: Brain/Biology, Personal Responsibility, Behaviour, and Environment. Each category contains specific factors with their respective importance ratings. For example, under Brain/Biology, genetic factors are rated by 12 as not important, 139 as important, and 89 as very important. Under Personal Responsibility, lack of willpower is rated by 44 as not important, 110 as important, and 86 as very important. The table provides a detailed breakdown of how different factors contributing to obesity are perceived by general practice nurses.

Navigate back to Table 1..

## Table 2. Long description

A table showing statistical analysis results of general practice nurses’ knowledge and perception of obesity care. The table has six rows and three columns. The columns are labeled ‘Question’, ‘P-value’, and ‘Result’. The rows present different questions asked to general practice nurses, their corresponding P-values, and the results indicating whether the training or BMI influences their responses. Row 1: Does having previous training make it more likely that general practice nurses accessed the MECC module? P-value: 0.002, Result: Yes. Row 2: Does having previous training make it more likely that general practice nurses accessed the ASOI Guidelines? P-value: 0.001, Result: Yes. Row 3: Does training influence general practice nurses rating of the importance of laziness in the development and/or progression of obesity? P-value: 0.024, Result: Yes. Row 4: Does training influence general practice nurses rating of the importance of laziness in the development and/or progression of obesity? P-value: 0.080, Result: No. Row 5: Does the BMI of a general practice nurse influence how they rate laziness? P-value: 0.430, Result: No. Row 6: Does the BMI of a general practice nurse influence how they rate lack of will power? P-value: 0.379, Result: No. Row 7: Do general practice nurses feel their BMI affects how patients with obesity view their advice? P-value: 0.001, Result: Yes.

Navigate back to Table 2..
