# Supplementary material for: Knowledge and perceptions of obesity care among general practice nurses in the Republic of Ireland
Source: Prim Health Care Res Dev. 2026 Jul 28;27:e80. doi: 10.1017/S1463423626101285 (PMC13439027; doi:10.1017/S1463423626101285)
Supplement: Supplementary file 1 [file S1463423626101285sup001.docx]

**Supplementary Material:**

Demographics, qualifications and body mass index of 240 general practice nurses who responded to a questionnaire on the knowledge and perceptions of obesity care among general practice nurses in the Republic of Ireland (2024)

| AGE OF PARTICIAPNTS | |  |
| --- | --- | --- |
| AGE | Number | % of Total |
| 25-29 | 2 | 0.5 |
| 30-39 | 36 | 15 |
| 40-49 | 97 | 40 |
| 50-59 | 81 | 34 |
| 60-69 | 23 | 10 |
| 70-79 | 1 | 0.5 |
| NUMBER OF YEARS WORKING IN GENERAL PRACTICE | | |
| YEARS | Number | % of Total |
| 0-4 | 64 | 27 |
| 5-9 | 57 | 24 |
| 10-15 | 31 | 13 |
| 15-20 | 41 | 17 |
| 20-25 | 29 | 12 |
| >25 | 18 | 7 |
| SINGLE DIVISIONS OF NMBI REGISTER | | |
| DIVISION | Number | % of Total |
| RGN only | 239 | 99.6 |
| RM only | 1 | 0.4 |
| MULTIPLE DIVISIONS OF NMBI REGISTER | | |
| RGN + | 130 | 54 |
| RGN+RM | 53 | 22 |
| RGN+RCN | 19 | 7.9 |
| RGN+PHN | 1 | 0.4 |
| RGN+RPN | 3 | 1.3 |
| RGN+RNP | 15 | 6.3 |
| RGN+RCN+RNP | 4 | 1.7 |
| RGN+RCN+RNT | 1 | 0.4 |
| RGN+RM+PHN | 2 | 0.8 |
| RGN+RM+RNP | 1 | 0.4 |
| RGN+RNID+RNP | 1 | 0.4 |
| RGN+RNP+cANP | 2 | 0.8 |
| RGN+RNP+RANP | 7 | 2.9 |
| BODY MASS INDEX | |  |
| BMI kg/m^2^ | Number | % of Total |
| <18.5 | 3 | 1 |
| 18.5-24.9 | 100 | 42 |
| 25-29.9 | 79 | 33 |
| 30-39.9 | 47 | 20 |
| <40 | 5 | 2 |
| Not given | 6 | 2 |
|  |  |  |
| Abbreviations: RGN: Registered General Nurse, RM: Registered Midwife, RCN: Registered Children’s Nurse, PHN: Public Health Nurse, RPN: Registered Psychiatric Nurse, RNP: Registered Nurse Prescriber, RNT: Registered Nurse Tutor, RNID: Registered Nurse in Intellectual Disability, cANP: Candidate Advanced Nurse Prescriber, RANP: Registered Advanced Nurse Prescriber, BMI: Body Mass Index, NMBI: Nursing and Midwifery Board of Ireland | | |

**Knowledge & Perceptions of Obesity Care among GPNs in the Republic of Ireland**

**Aug 04, 2024**

Consent

1. I have read and understood the Information Leaflet about this research project. I am satisfied that I understand the information provided and have had enough time to consider the information and understand what my participation in the research will involve.

I understand that participation in this study is voluntary and I am free to withdraw at any time, without giving a reason, at no disadvantage or penalty.

I am aware of the potential risks, benefits and alternatives of this research study.

I understand that the data collected during this study will be processed in accordance with data protection law as explained in the participant information leaflet.

I can access a copy of the Information Leaflet and this completed consent form for my records.

I consent to take part in this research study having been fully informed of the risks, benefits and alternatives.

I give informed explicit consent to have my data processed as part of this research study.

I understand that once I have submitted my response, it cannot be withdrawn, as it will have been anonymously submitted so cannot be separated from other submissions. *

Yes

No

Data Storage

2. *Storage and Future Use of Information (please tick only ONE option)* *

I give permission for my anonymous data to be stored for possible future research related to the current study only if consent is obtained at the time of the future research and the research is approved by a Research Ethics Committee

I give permission for my anonymous data to be stored for possible future research related to the current study without further consent being required but only if the research is approved by a Research Ethics Committee.

I request that all my data collected for this study can no longer be used by researchers in the future and that it be destroyed

General practice nurse profile & practice information

3. Are you employed as a Nurse in General Practice in the Republic of Ireland? *

Yes

No

4. What are your divisions on the Nursing and Midwifery Board of Ireland register? *

5. How many years have you been working as a nurse in general practice? *

The value must be a number

6. How old are you? *

The value must be a number

7. What is your BMI category (Optional)

under 18.5

between 18.5 and 24.9

between 25 and 29.9

between 30 and 39.9

40 or over

Knowledge of Obesity causes, identification & management

8. How important do you believe the following are in the development and/or progression of obesity? *

Not Important	Important	Very Important

Genetic factors

Metabolic factors

Endocrine Disorder

Addictive properties of food

Lack of willpower Laziness

Psychological/emotional problems

Not enough physical activity

Overeating

Poor nutritional knowledge

Eating out of the home, eg shops, takeaways, restaurants

Marketing of unhealthy foods

Low income

Stress

Poor quality or lack of sleep

Training in obesity causes, identification & management

9. Have you had training on obesity? Choose as many as you like. *

No

Webinars/conferences

Academic training

10. Have you completed the Making Every Contact Count Obesity Module on HSEland? *

Yes

No

Unaware of it

11. Have you accessed the ASOI Clinical Practice Guidelines on the Management of Obesity in Adults in Ireland? *

Yes

No

Unaware of them

Perceptions of GPNs on raising the topic about weight

12. **Are you comfortable having discussions with your patients about their weight?** *

Yes

No

13. When discussing obesity with a patient who mostly brings it up? *

Me

The Patient

14. What might stop you from starting a conversation about a person’s weight during an appointment? Choose as many as you like. *

They attend for a different reason

Lack of time

Obesity is not a medical issue

Fear of causing offense

Not sure what to say

Perceptions of GPNs towards obesity care

15. Please tick whether you agree or disagree with the following statements: *

Agree	Disagree

**Practice nurses have an essential role in identifying and treating people with obesity**

**I feel well prepared to manage people with obesity**

**Treatment for weight loss should be offered only to adults living with obesity**

**I do not believe I can have any effect on a patient's ability to lose weight**

**Practice nurses’ role is to refer people living with obesity to other professionals rather than attempt to treat them themselves**

**I feel my own weight affects how people living with obesity view my advice**

**For people living with obesity, even small weight loss can produce health benefits**

Role of the GPN in the assessment and diagnosis of obesity

16. In your own clinical practice, what tools do you usually use to assess obesity? Choose as many as you like. *

Assessment by eye

BMI

Waist/hip ratio

Waist circumference (measured in clinic)

Weight measured in clinic

Edmonson/Kings obesity staging score

Role of the GPN in the management of obesity care

17. In the treatment of obesity, I regularly discuss: *

Never	Sometimes	Often

Behavioural interventions

Pharmacological treatment

Bariatric/metabolic surgery

18. What factors would encourage you to consider discussing obesity pharmacotherapy treatments with your patient? Choose as many as you like. *

BMI over 25 with weight related co-morbidity

BMI over 30

BMI over 30 with weight related co-morbidity BMI over 40 with weight related co-morbidity

Inability to exercise

When the patient requests support

If required by relevant clinical guidelines/protocols

Barriers to GPNs providing obesity care

19. **What barriers do you experience to providing obesity care? Choose as many as you like.** *

Lack of training to discuss obesity management with patients

Lack of time in appointments

Lack of knowledge of obesity and obesity related conditions

Lack of clinical priority for treatment of obesity

Cost of treatments prohibit people living with obesity from accessing them

Shortage of specialists or specialist programmes to refer patients to

Lack of confidence in myself as role model

Resources to help GPNs provide obesity care

20. What resources would help you care for people living with obesity? Please choose a maximum of 3 options. *

Please select at most 3 options.

Recognition of obesity as a disease

Improved obesity referral process

Access to dieticians

Access to obesity management programmes

Access to living well programmes

Access to dedicated fitness programmes

Access to medication to help manage obesity

Increased GPN education related to the management of obesity
